# The Influence of Stomach Back-Shu and Front-Mu Points on Insular Functional Connectivity in Functional Dyspepsia Rat Models

**DOI:** 10.1155/2021/2771094

**Published:** 2021-09-14

**Authors:** Yuan Chen, Ying Zhao, Robert Yu-Sheng Tan, Pu-yue Zhang, Tao Long, Yu Shi, Hua-bin Zheng

**Affiliations:** ^1^Acupuncture and Tuina School, Chengdu University of Traditional Chinese Medicine, Chengdu, China; ^2^Department of Oncology, Chongqing Beibei Traditional Chinese Medical Hospital, Chongqing, China; ^3^Department of Acupuncture and Moxibustion, Affiliated Hospital of Chengdu University of Traditional Chinese Medicine, Chengdu 610072, China

## Abstract

Functional Dyspepsia (FD) is a common functional gastrointestinal disease, which can reduce the quality of life in patients. Prior research has indicated that insula is closely related to FD and that acupuncture can regulate the functional connectivity (FC) of FD. Therefore, we hypothesized that acupuncture on FD was effected through the insular pathway. To test our hypothesis, we performed electroacupuncture (EA) on FD rat models and then examined the FC between insula and other brain regions through resting-state functional magnetic resonance imaging (rs-fMRI). Seven-day-old male infant Sprague-Dawley (SD) rats were randomly divided into control group, FD model group, and FD acupuncture group, with twelve rats per group (*n* = 36). Upon establishing successful models, the FD acupuncture group was subjected to EA intervention using Stomach back-shu (BL-21) and front-mu (RN-12) points for ten consecutive days for durations of 20 minutes each day. After intervention, each group was subject to rs-fMRI. The digital image data obtained were analyzed using FC analysis methods. Subsequently, gastric ligation was performed to measure gastric emptying rates. Before EA intervention, the FD model group exhibited decreased functional connections between the insula and a number of brain regions. After EA intervention, FD acupuncture group exhibited increasing FC between insula and regions when compared to the FD model group, such as the primary somatosensory cortex (S1), hippocampal CA3 (CA3), polymorphic layer of dentate gyrus (PoDG), caudate putamen (CPu), and oral pontine reticular nuclei (PnO) (*P* < 0.05); decreasing FC was also exhibited between insula and regions such as the bilateral primary and secondary motor cortexes (M1/2), paraventricular hypothalamic nucleus (PVA), and limbic cortex (LC). These findings indicate that the effective treatment of FD using EA may be through regulating the abnormal FC between insula and several brain regions, in particular CA3, PoDG, and PVA.

## 1. Introduction

Functional Dyspepsia (FD) is a functional gastrointestinal disorder defined by the Rome IV consensus as the presence of symptoms thought to originate from the upper gastrointestinal region without organic causes; it can be divided into postprandial distress syndrome (PDS) and epigastric pain syndrome (EPS) [1]. Despite not being a life-threatening condition, it severely reduces quality of life for affected patients [2]. Current conventional allopathic treatment options exist for FD, such as *H. pylori* eradication, proton pump inhibitors (PPIs), and histamine-type-2-receptor antagonists (H2RAs); however, their efficacies are modest at best [3, 4], and they also result in negative side effects or negative long-term effects to the patient [5, 6]. For example, *H. pylori* eradication using antibiotics often produces gastrointestinal side effects such as diarrhea, nausea, taste distortion, stomatitis, and bloating, which reduce the tolerability of treatment and lead to treatment withdrawal [5]; treatments using PPIs have been shown to potentially cause serious long-term adverse effects, such as pneumonia, *C. difficile* diarrhea, risk of fractures, and hypomagnesemia [6]. Studies on the effects of H2RAs have typically suffered from poor quality or have shown a small effect on FD at best [4].

As an alternative form of treatment for FD that does not result in the same long-term side effects and resistance as drugs [7], acupuncture and/or electroacupuncture (EA) has been used in China for thousands of years to help relieve its various symptoms [8]. Research in this area has focused on the mechanisms behind both the pathogenesis of the condition [9] and the therapeutic effects of acupuncture on human and animal subjects [10–12]. Such studies often use imaging technologies such as positron emission tomography-computed tomography (PET-CT) [8, 9, 12] and functional magnetic resonance imaging (fMRI) [13–15]. Regardless of the type of imaging study, results have consistently shown that the insula, a brain region in the deep cerebral hemisphere involved in processing emotional responses, visceral sensation, interoceptive awareness, cognitive and affective regulatory functions, and olfactory and gustatory stimulation processing [9, 16–18], is an important area in FD research, since it is involved in both the pathogenesis and treatment of FD [8, 9, 12]. Studies involving human subjects in this area have shown weakened structural changes and functional connections of the insula in patients with FD [9], correlation between these changes and severity of FD symptoms [12], and changes in functional connections occurring through acupuncture or EA treatment of FD [8]. However, there has been little research into the activation pathways and mechanisms that are triggered through the insula when treating FD with acupuncture or EA. Furthermore, modeling of FD using animals has become an emerging area of research due to ethical or economic considerations arising from use of human FD patients.

Rats have commonly been used to mimic some of the disease outcomes and conditions of FD, such as psychological distress, delayed gastric emptying, impaired gastric accommodation, and visceral hypersensitivity, as described by Ye et al. [11]. Acupuncture point combinations such as the pairing of Stomach back-shu (BL-21) and front-mu (RN-12) points are among those typically used in acupuncture or EA treatment modalities of animal models [19, 20]. This combination has been shown specifically to improve decreased gastric motility through the hippocampus [21], dorsal vagal complex (DVC) [19, 20], or the paraventricular hypothalamic nucleus (PVA) [20]. However, like similar studies involving human counterparts, there has been little research into the activation pathways and mechanisms involving the insula in acupuncture or EA treatment of FD [21]. Due to the importance of the insula in both the pathogenesis and treatment aspects of FD, we hypothesize that it occupies a major role in the central mechanisms in acupuncture or EA treatment of FD. Our study aims to explore this hypothesis by conducting a seed-based functional connectivity analysis of FD rat models with EA intervention, in which the insula is designated as the region of interest (ROI).

## 2. Materials and Methods

### 2.1. Ethical Approval

This study was approved by the Experimental Animal Welfare and Ethics Committee of Chengdu University of Traditional Chinese Medicine (no. 2016-10). All efforts were made to minimize suffering.

### 2.2. Materials

The materials used are as follows: 0.1% iodoacetamide (Sigma Corporation, USA), isoflurane (ISO) (RWD Living Science Technology Inc., Shenzhen, China), Safe 2010 Safety Cabinet (Heto-Holten A/S, Denmark), PF Hetotherm Heating circulator (Heto-Holten A/S, Denmark), G6805-II electroacupuncture pulse generator (Qingdao Xinsheng Medical Instrument Factory, Shandong, China), Bruker BioSpec 70/20 USR small animal 7T MRI scanner (600 MHz) (Bruker Corporation, Germany), SPSS 20.0 (SPSS Inc., Chicago, IL, USA), and spmratIHEP based on the statistical parametric mapping (SPM8) software (Welcome Department of Cognitive Neurology, London, UK).

### 2.3. Animals and Grouping

Thirty-six healthy Sprague-Dawley (SD) rat pups (7 days old, day of birth = day 0) were used in this study (Certificate no.: CXK [Sichuan] 2015-030). The rat pups were randomly divided into control group (*n* = 12), FD model group (*n* = 12), and FD acupuncture group (*n* = 12) and placed in three separate cages. Although neonatal maternal separation has been shown to be one of the commonly used methods in producing FD animal models [11], it has also been identified as a risk factor in irritable bowel syndrome (IBS) [22]. To prevent the possibility of creating IBS models as a confounding factor, we placed one lactating female rat in each cage with sufficient food and water to provide feeding for the pups. After seven days of nursing to help rat pups acclimatize, the first phase of FD modeling was commenced.

### 2.4. FD Modeling

**Figure 1:**
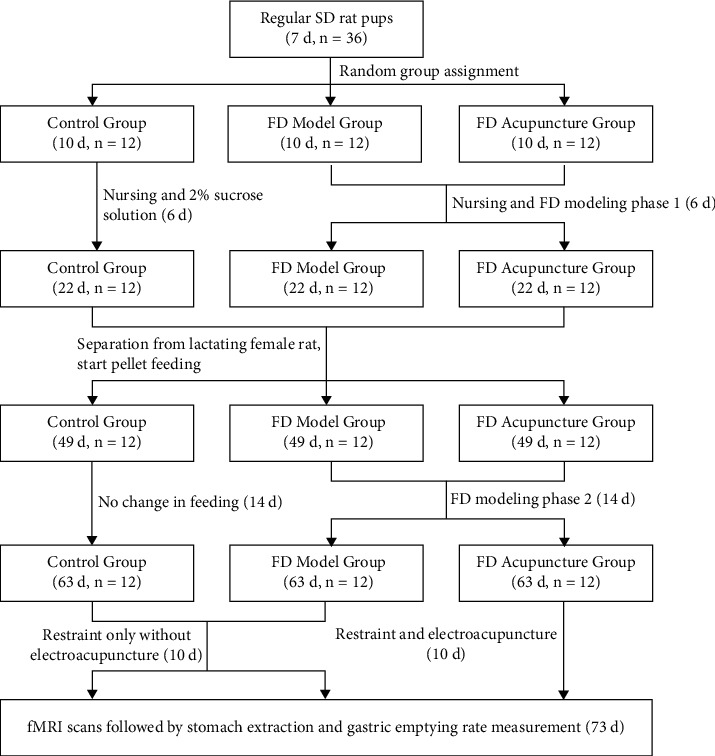
Experimental procedure.

A two-phase multiple-feature modeling method was used to create the FD model used in this study, including iodoacetamide-sucrose administration, tail clamping, and irregular feeding, which have been previously described [11, 21–24]. We aimed to produce FD rat models with the specific set of characteristics: presence of anxiety-like behaviour resulting in decreased appetite and water intake, reduction in activity, decrease of coat luster, increased knots in fur, and delayed gastric emptying. Phase one consisted of oral gavage administration of an iodoacetamide-sucrose solution, consisting of 0.1% iodoacetamide (IA) distilled water solution with a 2% sucrose distilled water solution, mixed at a ratio of 1 : 1 to induce neonatal gastric irritation [11]. The FD model and FD acupuncture groups received oral gavage administration of the IA-sucrose solution over 6 days, 0.2 mL per day, while the control group received oral gavage administration of 0.2 mL 2% sucrose distilled water solution over the same period, all alongside regular nursing [25]. After the six-day period, regular nursing was resumed until day 22, at which point the female rats were separated from the litters. From day 22 onwards, feed pellets were introduced until the rat pups reached 7 weeks of age, and we commenced phase two of FD modeling. Phase two consisted of tail clamping to induce stress and delayed gastric emptying [11, 26] and irregular feeding to disrupt regular feeding cycles [21] over a period of 14 days. Tail clamping provocation was implemented in the FD model and FD acupuncture groups by clamping sponge forceps at the distal 1/3 region of rat tails, without injury to the skin, for 30 minutes at a time, four times per day [27]. Discomfort from tail clamping made the rats irritable and anxious and caused infighting. Irregular feeding was also administered to the FD model and FD acupuncture groups during this period by withholding food (but not water) on odd-numbered days and feeding normally on even-numbered days [21]. The control group was fed normally during phase two without tail clamping. At the end of phase two on day 63, successful FD modeling was confirmed by rats in the FD groups having exhibited the desired model characteristics prior to the beginning of EA intervention. EA intervention was then administered from day 63 to day 73, followed by subsequent fMRI scanning and gastric extraction. Indirect indication of successful FD modeling was also confirmed after gastric extraction, when delayed gastric emptying rates were measured in the FD model group and compared to the control group, after EA intervention. The workflow of the experimental protocol is shown in Figure 1.

### 2.5. Acupuncture Point Location

**Figure 2:**
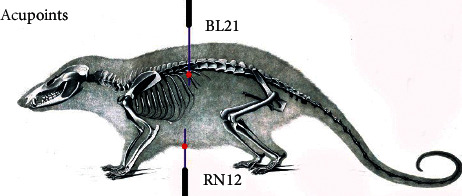
The locations of RN-12 and BL-21 acupuncture points on our rat model.

The Stomach front-mu Zhongwan (RN-12) and back-shu Weishu (BL-21) acupuncture points were located in the same manner as described by Wang et al. [19]. The location of Stomach front-mu point was established as being 20 mm above the umbilicus in the anterior centre line, needled at a depth of 2 mm [19]. The location of Stomach back-shu point was established as being 5 mm lateral to the twelfth thoracic vertebrae on both sides, needled at a depth of 4 mm [19].  Figure 2 shows the locations of RN-12 and BL-21 acupuncture points on our rat model [21].

### 2.6. Electroacupuncture Intervention

EA intervention commenced on day 63, when FD modeling had been successfully established. To prevent unnecessary struggle during the EA intervention, rats in all three groups were restrained, although only the FD acupuncture group received EA treatment. In addition, cotton cloth was used to cover the head of rats during the EA sessions to prevent unnecessary stress and to accommodate their preference for darkness, which better facilitated the EA procedures. The EA treatment administered to the FD acupuncture group consisted of needling both the RN-12 and one of BL-21 points (left and right sides, alternating on even and odd days) on rats sterilized with iodophor at the two point locations. Sterile one-time-use 0.25 × 13 mm needles were inserted at the previously indicated depth of 2 mm and monitored to avoid injury to the viscera. Due to the highly technical nature of EA treatment, all needle operators were licensed MDs who had been trained in acupuncture needling techniques. To avoid differences due to manual hand stimulation, needles were not stimulated by hand but were instead attached to an electroacupuncture apparatus (HANS-200A, Nanjing, China) to send controlled and uniformed stimulation to the needles (dilatational wave, frequency of 2/15 Hz, and output current of 1.5 mA). Treatment was administered for 20 minutes each day, beginning at 9 a.m. On rare occasions, rats who escaped from their restraints were rebound and had their treatments start from the beginning. The control and FD model groups, although also bound to eliminate differences in restraint-related stresses between the groups, were not subject to the EA treatment and were kept segregated from the FD acupuncture group to prevent undue stress triggered by noise from the FD acupuncture group.

### 2.7. fMRI Scanning, Observational Metrics, and Data Processing

At each phase of the study, metrics were collected with regard to changes in general observations, body weight, food intake, and water intake of the rats. The following criteria were observed and scored on a 20-point scale: coat color and luster, mental state, dexterity, body weight via digital scale, and amount of food and water intake [28]. Scoring was performed as follows and the changes were summarized using one-way analysis of variance (one-way ANOVA) as comparison:

1. Rat coat color and luster: 5 points for bright and shiny coat, 3 points for mildly bright and shiny coat, and 1 point for dry and dull coats
2. Rat dexterity: 5 points for extremely dexterous, 3 points for mildly dexterous, and 1 point for sluggish and not dexterous
3. Rat food intake: 5 points for no change in food intake, 3 points for slight changes in food intake, and 1 point for significantly reduced food intake
4. Rat body weight: 5 points for increased body weight, 3 points for unchanged body weight, and 1 point for significantly reduced body weight

After EA intervention, at least 6 rats were randomly chosen from each group according to the requirements of imaging research, and 7.0T animal rs-fMRI scans were performed on these selected rats. The scanning method was to extract 2-3 EPI sequences for each rat and 1 RARE sequence (EPI sequence: TE = 16 ms, TR = 1000 ms, segments = 2, average = 1, repetition = 300, FOV = 25 × 25 mm^2^, Matrix = 80 × 80, slice = 18, and thickness = 0.75 mm; T2 structural image: RARE sequence, TE = 12 ms, TR = 4000 ms, average = 4, FOV = 25 × 25 mm 2, Matrix = 256 × 256, slice = 18, and thickness = 0.75 mm). The data obtained was processed using SPSS 20.0 statistical software package.

**Figure 3:**
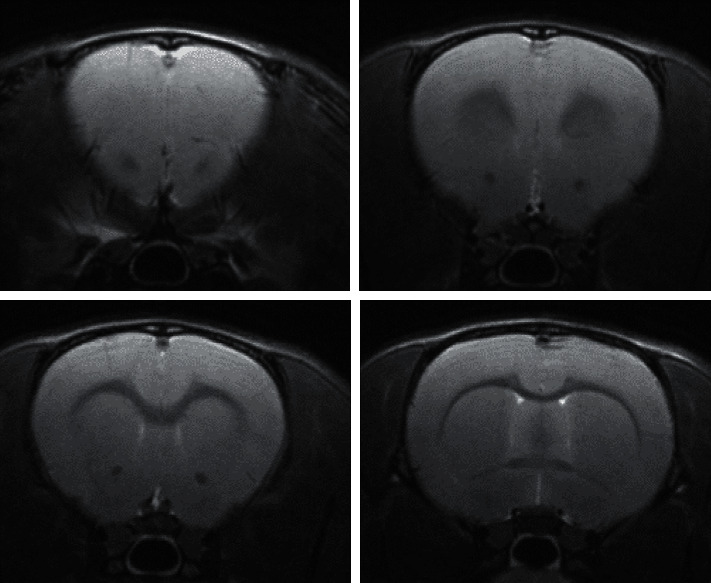
Example of T2 structural phases.

After data collection was completed, spmratIHEP toolkit based on the statistical parametric mapping (SPM8) software was used to sequentially perform image preprocessing, brain segmentation, data extraction, and functional connectivity calculation of the brain. Establishing the insula-related ROI as the seed in a similar manner as described by Nie et al. [29], its functional connections were compared to those of other voxels of the entire brain in all three of the control, FD model, and FD acupuncture groups. Through nonparametric tests, it was found that when *P* < 0.05, clusters greater than 6 voxels were considered statistically significant. Permutation TFCE0.05 was used for correction. After correction, the statistically significant brain activation area image was superimposed onto the standard brain image. After statistical calculations were made, the positions, position coordinates, and signal types of the activated brain areas were recorded (see Figure 3 for an example of T2 structural phase).

### 2.8. Detection of Gastric Emptying Rate of Rats in Each Group after Intervention

Subsequent to fMRI scanning, gastric emptying rate was measured in the scanned rats, using methods similar to those described by Francis et al. and Asuzu and Njoku [30, 31]. On the last day of EA intervention, 6 rats from each group were randomly selected and placed into the anesthesia box, where a mixture of 5% isoflurane and 100% oxygen gas was administered through inhalation. 50 minutes prior to anesthesia, rats were also fed starch paste of 1 g/ml. Once anesthetized, the isoflurane concentration was reduced to 3% to maintain the anesthesia state. Then the rats were fixed, and a surgical scalpel was used to dissect the peritoneal cavity, which was opened gently using sterile gauze.

The cardia and pylorus were quickly ligated with surgical sutures, and the entire stomach was subsequently removed. The stomachs were weighed digitally after surface mucosa and excess tissue were removed and excess blood and fluids were wiped clean from the surface. After recording the gross stomach weights, small animal surgical shears were used to cut along the greater curvature of the stomach, with the remainder starch paste being washed out using a saline solution. The stomach was subsequently dried with gauze and weighed to determine net stomach weight. Upon recording net stomach weight, the gastric emptying rate was calculated as follows: (1 − (gross stomach weight − net stomach weight)/starch weight) × 100%. Rats were then euthanized via anesthesia.

### 2.9. Statistical Analysis

Statistical analyses were performed using SPSS 20.0 software. Scoring of general observations, rat body weight, changes in food intake, changes in water intake, and gastric emptying rate are represented as the mean ± SD. The data of all three groups formed positive distributions, and the groups were compared using one-way analysis of variance (ANOVA) and Kruskal-Wallis H tests. Brain functional connection results were analyzed using nonparametric test. When the *P* value was less than 0.05 and the number of clusters was more than 6, the difference was considered statistically significant. Permutation TFCE 0.05 was used for correction.

## 3. Results and Discussion

### 3.1. Observations regarding General State

**Figure 4:**
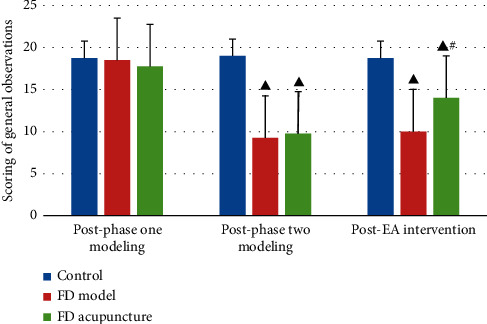
Scoring of general observations in different groups; data are presented as mean ± SD (*n* = 6). Note: ^▲^*P* < 0.05, versus control group; ^#^*P* < 0.05, versus FD model group.

Changes in coat color and luster, mental state, dexterity, body weight, and amount of food and water intake are summarized in Figure 4 using one-way analysis of variance (one-way ANOVA) as comparison.

### 3.2. Body Changes in Rat Body Weight in Each Phase

**Figure 5:**
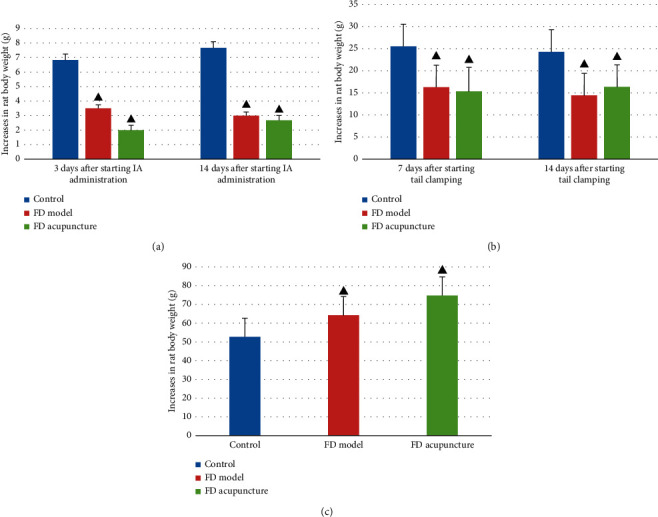
Increases in rat body weight in different groups. (a) During phase-one oral gavage administration; (b) during phase-two tail clamping; (c) during EA intervention. Data are presented as mean ± SD (*n* = 6). Note: ^▲^*P* < 0.05, versus control group.

Body weight for each rat was measured using a digital scale prior to FD modeling, during each phase of FD modeling, and after EA intervention. During phase-one oral gavage administration, weight was recorded on days 1, 3, and 6. The control group had statistically significantly higher body weight gain compared to both FD groups (*P* < 0.05). Both FD groups maintained similar weight throughout phase one (*P* > 0.05). Changes were compared using one-way ANOVA and are summarized in Figure 5(a).

During phase-two tail clamping, weight was recorded on days 1, 7, and 14. Throughout the tail clamping process, both FD model and FD acupuncture groups maintained similar increases (*P* > 0.05), which were significantly less compared to the control group (*P* < 0.05). Changes were compared using one-way ANOVA and are summarized in Figure 5(b).

During EA intervention, weight was recorded on the first day and last day. The FD acupuncture group had the highest increase in weight, followed by the FD model group and control group. The weight gain for FD acupuncture group was statistically significant compared separately to both the FD model and control groups (*P* < 0.05). The weight gain for FD model group was statistically significant compared to the control group (*P* < 0.05). Changes were compared using one-way ANOVA and are summarized in Figure 5(c).

### 3.3. Changes in Food and Water Intake of Rats in Each Group

Rats were fed each day at 9 a.m. with a fixed amount of feed that was weighed on a digital scale. The amount of remaining feed was measured at 9 a.m. the next morning, and the difference was taken as the change in daily food intake. Water intake was measured in a similar manner as the change in daily water intake.

**Figure 6:**
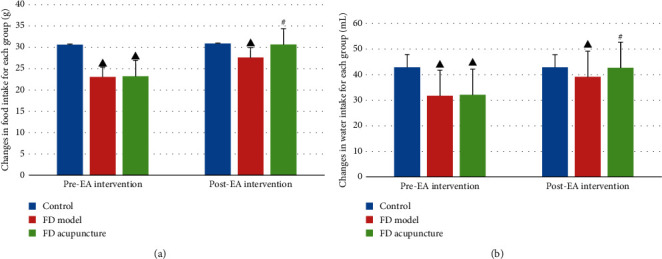
(a) Comparison of changes in food intake before and after EA intervention; (b) comparison of changes in water intake before and after EA intervention. Data are presented as mean ± SD (*n* = 6). Note: ^▲^*P* < 0.05, versus control group; ^#^*P* < 0.05, versus FD model group.

Prior to EA intervention, both FD groups exhibited less food and water intake compared to the control group (*P* < 0.05). After EA intervention, both FD groups exhibited greater amounts of food and water intake, although FD model group was still less than that of FD acupuncture (*P* < 0.05). The results were compared using one-way ANOVA and are summarized in Figures 6(a) and 6(b).

### 3.4. Gastric Emptying Rate after EA Intervention

**Figure 7:**
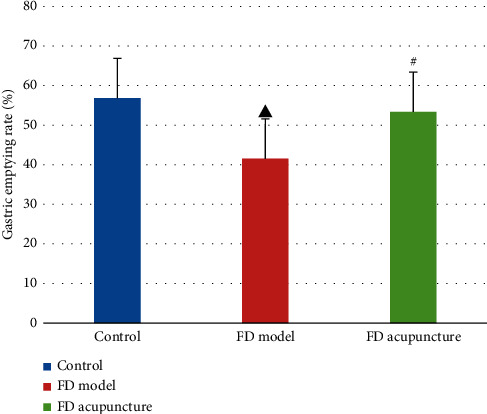
Comparison of gastric emptying rates between groups (*n* = 6). Note: ^▲^*P* < 0.05, versus control group; ^#^*P* < 0.05, versus FD model group.

A summarized comparison of the gastric emptying rates between the groups after EA intervention is listed in Figure 7 with comparisons performed using the Kruskal-Wallis H test. The FD model group had a significant lower gastric emptying rate when compared to the control group (*P* < 0.05), while the FD acupuncture group had a significantly higher gastric emptying rate when compared to the FD model group (*P* < 0.05).

### 3.5. Rat fMRI Imaging Results

**Figure 8:**
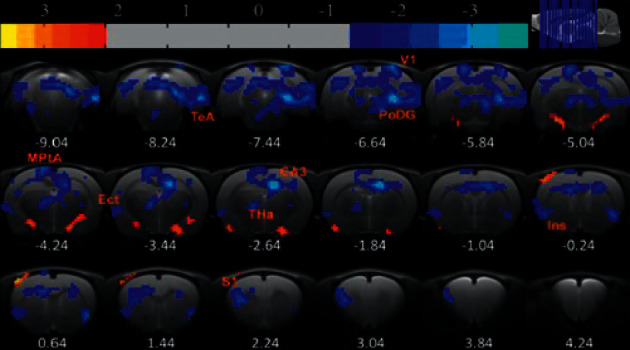
Regions exhibiting significant functional connections with the insula, FD model group after EA intervention.

**Figure 9:**
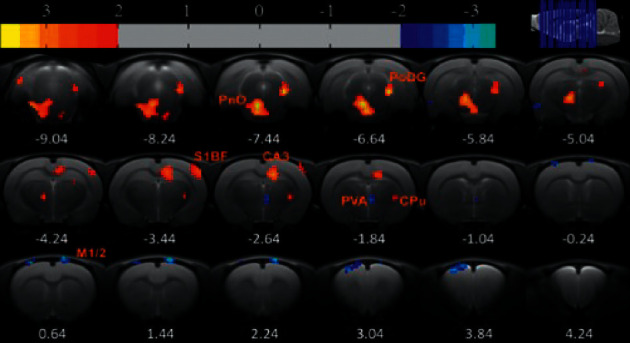
Regions exhibiting significant functional connections with the insula, FD model group after EA intervention.

Six rats were chosen from each of the three groups, and rs-fMRI scanning was performed after EA intervention. The scans established the insula as the seed while exploring its functional connections with the rest of the brain:

1. Compared to the control group, the FD model group contained regions which had significant functional connections with the insula, as seen in Table 1 and Figure 8. Compared with the control group, rats in the FD model group exhibited decreased functional connections in regions including the primary somatosensory cortex, hippocampal CA3, thalamus, ectorhinal cortex, polymorphic layer of dentate gyrus, medial parietal association cortex, primary visual cortex, and temporal association cortex (*P* < 0.05, cluster >6, differences are statistically significant).
2. After EA intervention, when compared to the FD model group, the FD acupuncture group displayed regions which had significant functional connections to the insula, summarized in Table 2 and Figure 9. The regions which exhibited increased functional connections include caudate putamen, barrel field of primary somatosensory cortex, hippocampal CA3, oral pontine reticular nuclei, and polymorphic layer of dentate gyrus (*P* < 0.05, cluster >6, difference is statistically significant); some regions also exhibited decreased functional connections, including the paraventricular nucleus of hypothalamus, bilateral primary motor cortexes, and limbic cortex.

### 3.6. Discussion

Present explanations on the mechanism of acupuncture center around neural mechanisms or humoral mechanisms; thus our exploration focuses on the neural mechanism of acupuncture in the treatment of FD. There is increasing evidence in support of the presence of abnormal central changes of FD patients in addition to the peripheral changes in gastrointestinal tract. Previous results have suggested that the frontal cortex, somatosensory cortex, insula, ACC, thalamus, hippocampus, and amygdala were activated in FD patients prior to treatment, with the insula in particular occupying a key role [9, 32]. Interestingly, several studies have indicated that the brain regions activated in FD patients at baseline are different than those with acupuncture or EA intervention [8, 10]. This phenomenon suggests that the central mechanism involved in the pathogenesis of FD may be different than that of acupuncture treatment of FD patients and suggests the possibility of different pathway(s) in acupuncture treatment of FD patients, which we also explore in our results.

Our current rs-fMRI study involving seed-based functional connectivity analysis on EA treatment of FD rat models establishing the insula as ROI has revealed the following: (a) the combination of RN-12 and BL-21 acupoints can be used to improve gastric motility, which confirms the results of previous studies [19–21], and, more importantly, (b) specific patterns of increasing and decreasing functional connectivity not only exist with regard to the insula in FD rat models but also occur in EA intervention of FD.

Initially, we created a two-phase, multiple feature rat model of FD using methods previously established, including neonatal gastric irritation through oral gavage administration of iodoacetamide, tail clamping, and irregular feeding [11, 21, 23]. Successful models of both FD model and FD acupuncture groups were confirmed through monitoring of decreasing point measurements of general observations, increased anxiety-like behaviour, and infighting, resulting in decreased body weight and food and water intake, and through delayed gastric emptying rates which were measured subsequent to rs-fMRI scans. All measures that were recorded, except for gastric emptying rates, showed that statistically significant differences existed between the two FD groups and the control group. Our results also showed no statistically significant differences amongst the two FD groups prior to EA intervention, which confirmed successful FD modeling as reported in other studies [11, 21, 23]. Throughout the EA intervention phase of the study, the data collected regarding weight gain and food and water intake of the FD model group revealed two points of interest which seemed to contradict the initial effects of FD modeling to produce decreased weight gain, food intake, and water intake. First, the FD model group exhibited more weight gain than the control group throughout the EA intervention phase, even though FD model group was only restrained and not subject to EA intervention, as shown in Figure 5(c). Second, the FD model group also exhibited significant increases in food and water intake throughout the EA intervention phase, despite not being subject to EA intervention, as shown in Figures 6(a) and 6(b). These changes were unexpected because we initially believed that the FD modeling measures employed would produce permanent pathophysiological changes such as permanent increases in weight loss as noted by Wu et al. [23]. However, similar trends were shown in another study by Zhang et al. measuring the effects of Shen-Ling-Bai-Zhu-San on FD rat models [33], where the FD model group exhibited increases in food and water intake without intervention. Here, the data seemed to show other factors at work which decreased the effects of FD modeling on the FD model group, despite this group not being subject to EA intervention. Since no other interfering measures were applied to the FD model group, we hypothesize that these improvements may have had to do with the natural healing functions of the body and take note of this as a point of interest to consider in future research.

After EA intervention and the rs-fMRI process, gastric ligation was performed to both indirectly confirm successful modeling and measure the effect of EA intervention on gastric emptying rates. We found that the control group had the highest gastric emptying rate (56.82%), followed by the FD acupuncture group (53.39%) and then followed by the FD model group (41.54%), which confirmed the results of previous research, indicating that EA treatment using the combination of RN-12 and BL-21 acupoints improves gastric motility [21]. Interestingly, despite previous research by Li et al., which showed that EA at RN-12 tended to suppress gastric motility in human subjects when used by itself [34], our results showed that the opposite was true. We hypothesize that the difference in outcome may be related to the synergistic effects of the BL-21 and RN-12 acupoint combination, as opposed to just using the RN-12 acupoint by itself.

In the brain imaging process, we conducted a seed-based functional connectivity analysis of the FD model and FD acupuncture groups after EA intervention, establishing the insula as ROI.

#### 3.6.1. Functional Connections in FD Model Group and Comparisons to Previous Results

In the FD model group, significantly decreased functional connections were found only in the following brain regions: S1, CA3, THa, Ect, PoDG, MPtA, V1, and TeA, which bear both similarities and contrasts to previous results. Our results are similar to previous findings that indicate abnormal functional connections found between insula and S1, hippocampal CA3, and PoDG regions. In a systematic review of sixteen studies of FD patient brain activity by Lee et al., abnormal brain activity was frequently found in several areas across resting-state FD patients, including the insula, hippocampus, S1, prefrontal cortex, anterior cingulate cortex, and amygdala [32].

Activation in S1 has been reported in the literature previously. In a PET-CT study on FD patients by Zeng et al., S1 activity was shown in FD patients treated with acupuncture, though not in FD model patients [10]. Similar results were observed in a review by Lee et al. in terms of functional abnormalities of FD patients in several brain regions including S1 [32]. Findings from the same review also indicate that the temporal lobe and S1 were commonly activated during simulated distension of FD rat models using balloons [32]. Since one of the main symptoms of FD is epigastric pain, which may be accompanied by distension, the activation of S1 in our results could be interpreted as another measure of FD modeling success.

Both CA3 and PoDG are located within the hippocampus, and previous results in an H_2_^15^O PET study by Van et al. have shown that the hippocampus is activated in resting-state FD patients with history of physical abuse [35]. Another FD study reported that mRNA expression of GHS-R in hippocampus increased in the EA group compared with the model group [36]. In our study, the tail clamping and irregular feeding phases have been shown to create stress in rats and could have contributed to similar activation in the hippocampus.

Similar to the decreasing functional connections found between insula and THa within our results, a previous fMRI study of FD patients with acupuncture intervention by Liu et al. also found decreased functional connectivity in the right THa with the right anterior insula in resting-state FD patients [9]. An ^18^F-FDG PET-CT study by Zeng et al. showed deactivating THa activity in FD patients treated with acupuncture but not in resting state [8]. However, the discrepancies in THa activity were also observed by Liu et al. in another study investigating neural patterns in FD patients without acupuncture intervention, while their explanation for the differences was that dysfunctions in visceral sensory pathway can affect the “switchboard” ability of the THa to coordinate multiple actions [14]. Likewise, similar discrepancies exist for the decreasing functional connections for the TeA region found in our study compared to results of previous studies. The aforementioned fMRI study with acupuncture intervention by Liu et al. found decreased functional connectivity of the right anterior insula with right THa, and FD patients had negative correlation between disease duration and the functional connectivity of right anterior insula with THa [9]. However, another fMRI study by Fang et al. showed activity in the inferior temporal gyrus in FD patients prior to acupuncture treatment [13], while the neural pattern study by Liu et al. found resting-state activity in both the THa and temporal pole [14]. The explanation of discrepancies by Liu et al. in their neural pattern study was that the temporal pole was related to the neuropsychological mechanism of emotion in FD patients and that patients with different disease durations could exhibit differing activity in this region [14]. In our study, the duration of disease was uniform among the FD model and FD acupuncture groups, leading us to reject the disease duration explanation for rat models. Instead, we believe that the activation of the TeA region in our results may be due to recall of fear in the rat models, as suggested by Cho et al. in their study on activation of the TeA from remote recall of fear [37].

Activities in V1, MPtA, and ectorhinal regions from our results were not observed in other studies. Increasing functional connectivity in V1 had been observed in a previous fMRI study of healthy subjects treated via acupuncture by Dhond et al., although their study did not focus on FD patients [38]. The MPtA region has been shown to be associated with the processing of spatial tasks in rats [39]. Furthermore, the ectorhinal complex has been shown to relate to the freezing of movements in conditioned fear in rats [40], and its activation may be due to similar reasons as our proposed explanation of TeA activation. Due to limited availability of research, further study of these brain regions is required.

#### 3.6.2. Functional Connections in FD Acupuncture Group and Comparisons to Previous Results

In the FD acupuncture group, a different pattern of both significantly increasing and decreasing functional connections is seen in the following brain regions: left and right M1/2, PVA, CPu, S1BF, CA3, PRh, PnO, and PoDG.

In contrast to the FD model group, functional connections increased between the insula and the regions of S1, hippocampal CA3, and PoDG. Changes in these same regions were found in one study reviewed by Lee et al., indicating that brain activity increased in S1 but decreased in the insula and hippocampus in FD patients after 20 EA sessions [32]. Besides these similarities, we also noticed differences in comparison to results from another fMRI study by Zeng et al. [8], in which functional connectivity was measured between various ROIs in human FD patients before and after acupuncture treatment, in that they did not find functional connections between the anterior insula and hippocampus regions. The hippocampus brain region was previously shown by Wang et al. to be related to the regulation of gastric motility, as EA of RN-12 and BL-21 resulted in reduction of glutamate and subsequently improved gastric mobility [21]. The specific hippocampal CA3 and PoDG regions not only form part of the hippocampal formation [41] but also are involved in memory [38, 42, 43], production of new neurons after injury [44], the healing of physical trauma [45], and the remodeling of synapses after injury, all of which are functions that may help explain the treatment efficacies of EA or acupuncture towards FD. We attribute the discrepancies between our results and those of Fang et al. to differences in methodology, including different acupuncture points used, and differences in manual acupuncture versus EA. Furthermore, our findings would appear to suggest that abnormal functional connections between the insula and the hippocampal CA3 and PoDG regions form part of the mechanism which EA regulates in treating FD.

With regard to increased functional connectivity between the insula and CPu, S1BF, and PnO regions, research has shown that functional connections decreased between the insula and CPu in alcohol-dependent rats going through early withdrawal [46]. Both the insula and S1BF have also been shown to belong to the circuit which regulate gustatory information [47], and connections between the insula and PnO are yet to be explored, with more research required in these areas overall.

In contrast to the FD model group, functional connections decreased in the regions of left and right M1/M2, PVA, and PrH. PVA of the hypothalamus region is of particular interest, as previous studies, including a PET-CT brain imaging study by Zeng et al., have shown that the hypothalamus, insula, and ACC have been considered as key regions of the brain-gut axis [8] and that activations of these areas have been shown in studies for most gastrointestinal diseases [48]. The hypothalamus PVA has been shown in previous studies to play a role on its own in the regulation of appetite through the release of Neuropeptide Y (NPY) [49, 50], as well as in the regulation of thirst [51]. Functional connections between the insula and PVA were not exhibited in the FD model group but were exhibited in the FD acupuncture group. Therefore, the connection between insula and PVA may be part of the mechanism which improves food and water intake as part of acupuncture treatment of FD. Together with the results related to the hippocampus region discussed earlier in this paper, our results suggest that functional connections between the insula and the CA3, PoDG, and PVA regions are regulated through EA treatment in FD.

The functional connection results of the FD model (Table 1) and FD acupuncture groups (Table 2) indicate that different brain regions are activated in the two groups and that the only common active regions in the two groups are CA3 and PoDG of the hippocampus. Even though the importance of the CA3 and PoDG regions had been discussed earlier in this section, the observation of differing brain regions between two groups warrants attention. While it may be simple to hypothesize that the functional connections between the insula and brain regions in FD model patients would be reversed after EA intervention, both our results and those of prior studies have shown that this is not the case. One reason for this difference may be due to the way that compensatory strategies are used in healing of the brain. Hylin et al. examine the healing of brain injuries in their review and suggest that the brain is able to intrinsically react to change by inducing new growth pathways which help neurons near and distant from the affected area to survive, repair, and form new connections in order to achieve compensation or recovery [52]. Likewise, it is possible that EA intervention in our FD acupuncture group could contribute to a similar effect through compensation, thus resulting in the differences in brain regions, and this could be an area of research for future studies.

#### 3.6.3. Limitations

With regard to limitations, although our results indicated functional connections between the insula and different brain areas in FD rats using fMRI technology, they do not provide insight into chemical composition of the definite pathways between the insula and these brain regions. This latter consideration was outside the scope of our current study, and so we did not include it in our submission but is part of ongoing trials in our research group. In order to further explore the key role of the insula in the central mechanism behind EA efficacy of gastrointestinal regulation, our research group used the same method of FD rat modeling for analysis and concentrations of important metabolites in the insula between control, FD model, and FD acupuncture groups through magnetic resonance spectroscopy (MRS) instead of fMRI, so as to analyze the chemical composition of our suggested central mechanism [53]. The results from Long showed that, after EA treatment using stomach front-mu and back-shu points, concentrations of N-acetylaspartate (NAA), aspartic acid (Asp), and glycine (Gly) decreased significantly. These results suggest changes in chemical composition of the insula in FD rats after EA treatment and serve as a preliminary investigation into the chemical composition of the insular pathway in the treatment of FD.

## 4. Conclusions

In conclusion, our study aimed to explore the role of the insula in the EA treatment of FD through rs-fMRI seed-based functional connectivity analysis. The results showed the following: (a) in our two-phase multiple feature rat FD models created using iodoacetamide-sucrose administration, tail clamping, and irregular feeding, EA intervention using RN-12 and BL-21 successfully improved several measures in our rat FD models, including general observations, increased food and water intake, and improved gastric motility and, (b) after EA intervention, a number of both increasing and decreasing functional connections were seen between the insula and a number of brain regions, in particular the hippocampal CA3 and PoDG regions which are related to injury healing, and the hypothalamus PVA region which regulates thirst and appetite, and these may be parts of the central mechanism through which EA expresses its efficacy in the treatment of FD. One significant question raised in our study is that although the insula was shown to play a role in EA treatment of FD, both the magnitude and exclusiveness of its role are unknown. A potential subsequent step in this area of research would be to try and determine the magnitude and exclusivity of the role of the insula by blocking insular cortex function in rat FD models and then observing whether there are changes in the efficacy of EA intervention to see whether other action mechanisms are at work.

## Figures and Tables

**Table 1:** FC results (control group versus FD model group).

Brain region	Brain functional connection results		Standard deviations	
	Voxel size	*t* value	Mean ± SD (control)	Mean ± SD (FD model)
Primary somatosensory cortex (S1)	133	−3.4199	0.16 ± 0.11	0.06 ± 0.06
Hippocampal CA3 (CA3)	>200	−4.9154	0.23 ± 0.09	0.10 ± 0.08
Thalamus (THa)	>100	−3.1142	0.10 ± 0.11	0.02 ± 0.06
Ectorhinal cortex (Ect)	35	−2.6699	0.25 ± 0.12	0.16 ± 0.09
Polymorphic layer of dentate gyrus (PoDG)	>100	−4.4994	0.13 ± 0.08	0.02 ± 0.06
Medial parietal association cortex (MPtA)	>100	−3.2009	0.22 ± 0.16	0.09 ± 0.09
Primary visual cortex (V1)	>100	−3.9261	0.16 ± 0.12	0.06 ± 0.08
Temporal association cortex (TeA)	>100	−4.4293	0.19 ± 0.11	0.07 ± 0.09

In the FD model group, regions exhibit significant functional connections to the insula. Negative *t* values denote weakening connection, while positive *t* values denote strengthening connections.

**Table 2:** FC results (FD model group versus FD acupuncture group).

Brain region	Brain functional connection results		Standard deviations	
	Voxel size	*t* value	Mean ± SD (FD model)	Mean ± SD (FD acupuncture)
Right primary and secondary motor cortexes (M1/2)	44	−2.909	0.13 ± 0.09	0.05 ± 0.10
Left primary and secondary motor cortexes (M1/2)	23	−3.3159	0.09 ± 0.05	0.04 ± 0.07
Paraventricular nucleus of hypothalamus (PVA)	13	−2.7523	0.11 ± 0.08	0.05 ± 0.09
Caudate putamen (CPu)	9	2.56917	0.10 ± 0.05	0.14 ± 0.07
Barrel field of primary somatosensory cortex (S1BF)	29	2.0751	0.12 ± 0.07	0.19 ± 0.09
Hippocampal CA3 (CA3)	60	3.1749	0.07 ± 0.09	0.16 ± 0.11
Oral pontine reticular nuclei (PnO)	257	3.6603	−0.01 ± 0.06	0.09 ± 0.10
Polymorphic layer of dentate gyrus (PoDG)	62	3.5844	0.04 ± 0.07	0.11 ± 0.10

In the FD acupuncture group, regions exhibit significant functional connections to the insula. Negative *t* values denote weakening connection, while positive *t* values denote strengthening connections.

## Data Availability

The data used to support the findings of this study are available from the corresponding author upon request.
